# Tualang Honey Has a Protective Effect Against Photodamage and Skin Cancer: An In Vivo Study

**DOI:** 10.3390/nu16244314

**Published:** 2024-12-13

**Authors:** Mohammed Asif Sherwani, Erin M. Burns, Israr Ahmad, Ahmed Omar Jasser, Ariq Chandra, Nabiha Yusuf

**Affiliations:** Department of Dermatology, Heersink School of Medicine, University of Alabama at Birmingham, Birmingham, AL 35294, USA; sherwani@uab.edu (M.A.S.);

**Keywords:** tualang honey, ultraviolet radiation, DNA damage, inflammation, skin cancer

## Abstract

Background/Objective: Ultraviolet (UV) B radiation leads to DNA damage by generating cyclobutane pyrimidine dimers (CPDs). UVB-induced CPDs can also result in immune suppression, which is a major risk factor for non-melanoma skin cancer (NMSC). UVB-induced CPDs are repaired by nucleotide repair mechanisms (NER) mediated by xeroderma pigmentosum complementation group A (XPA). The purpose of this study was to investigate the use of TH as a chemopreventive agent against the development of skin cancer. Method: SKH-1 hairless mice were exposed were fed with TH (0.1% *v*/*v*) for two weeks and exposed to a single dose of UVB (180 mJ/cm^2^). Dorsal skin was harvested 24 h post-UVB exposure for evaluation of DNA damage and repair. Lymph nodes were also harvested to prepare single cell suspension for flow cytometric evaluation. For carcinogenesis experiments, SKH-1 hairless mice were given TH (0.1% *v*/*v*) ad libitum and exposed to UVB (180 mJ/cm^2^) thrice a week for 30 weeks. Results: Feeding SKH-1 hairless mice with TH (0.1% *v*/*v*) for two weeks prior to a single dose of UVB (180 mJ/cm^2^) led to a significant increase in XPA in skin and DNA repair cytokines IL-12 and IL-23 in draining lymph nodes. Furthermore, when subjected to the photocarcinogenesis protocol; mice fed with TH developed significantly fewer tumors in comparison to mice fed on drinking water. Conclusions: Our data demonstrate that TH has a protective effect against UVB-induced DNA damage, immune suppression, and skin cancer. Future studies will further investigate the potential of TH as a preventive treatment for NMSC.

## 1. Introduction

Current epidemiological data indicate that more than two million people in the United States develop nonmelanoma skin cancers every year [1]. Ultraviolet (UV) B radiation (290–320 nm) induces DNA damage, which plays a key role in suppressing immune responses and initiating non-melanoma skin cancers [2,3,4]. UVB-radiation-induced DNA damage includes alterations in genes involved in stress response, cell cycle control, DNA synthesis, and DNA repair [5]. Incorrect or inefficient repair of DNA damage ultimately leads to the introduction of deleterious mutations after DNA replication, resulting in initiation of non-melanoma skin cancers [6,7]. In addition to generating mutant cells, UVB also disrupts the host immune responses that have evolved to recognize and eliminate these cells before they progress into clinically evident malignancies [3,4].

UVB-induced DNA damage is one of the earliest molecular events in the development of UVB-induced immune suppression [8,9]. The cytokine interleukin (IL)-12 stimulates the expression of DNA repair enzymes and augments the repair of UVB-damaged DNA [10]. IL-12 also stimulates the induction of T-helper type 1 cell-mediated immune responses, including those against UVB-induced tumors [10,11]. IL-23 has also been reported to repair UVB-induced cyclobutane pyrimidine dimers (CPDs) and prevent immunosuppression [12].

The intake of photochemopreventative agents to protect against the adverse consequences of UVR has gained attention. Previous studies have cited the protective nature of polyphenols and phenolic acids found in naturally occurring substances and food products [13]. One such compound that shows promise is tualang honey (TH). Honey is not ubiquitous, and can differ in not just chemical composition (phytochemicals, carbohydrates, volatile constituents), physical properties (color, viscosity, hygroscopic properties, and pH), and taste, but also in their different biological activities [14]. TH is a Malaysian honey that has been studied for its antioxidant properties. The proposed components responsible for the oxidation-reduction properties of TH are phenolic acids, flavonoids, water-soluble vitamins, and various enzymes. Unlike other types of honey, TH is rich in phenolic acids, flavonoids, and various enzymes, which confer potent antioxidant, anti-inflammatory, and immunomodulatory effects [14]. These bioactive compounds are hypothesized to enhance DNA repair and modulate immune responses, making TH a promising candidate for photochemoprevention. While other types of honey have been investigated for their health benefits, TH’s distinct composition and higher radical scavenging activity set it apart, justifying its selection for this study.

Our previous work with TH demonstrated the protective effects against UVB-induced inflammation and DNA damage in a mouse keratinocyte cell line, with study results showing that TH inhibited UVB-induced DNA damage and inflammatory cytokines, and enhanced repair of CPDs, amongst many other anti-inflammatory parameters measured [15].

Despite the growing interest in natural chemopreventive agents, there is a lack of comprehensive research investigating the protective effects of TH against UVB-induced DNA damage, inflammation, and skin carcinogenesis. The existing studies on TH primarily focus on its antioxidant properties in vitro, leaving a significant gap in understanding its in vivo efficacy and mechanisms of action in the context of skin cancer prevention.

The current study aims to address this gap by evaluating the potential of TH as a photochemopreventive agent using an established murine model of UVB-induced skin damage and carcinogenesis. SKH-1 hairless mice were used for their susceptibility to UVB-induced skin changes, mimicking human skin responses. Mice were supplemented with TH and subjected to UVB exposure to assess DNA repair, cytokine expression, inflammatory responses, and tumor development. Specific assays, including immunohistochemistry, flow cytometry, and ELISA, were employed to quantify CPDs, cytokine levels, and inflammation, providing a detailed analysis of TH’s protective mechanisms.

This study aims to establish tualang honey as a natural, safe, and effective dietary intervention for preventing UVB-induced skin damage and carcinogenesis, filling a critical gap in the current understanding of natural photochemopreventive agents.

## 2. Material and Methods

### 2.1. Animals and Reagents

Tualang honey used in this study was provided by the Federal Agricultural Marketing Authority Malaysia (FAMA), Malaysia. SKH-1 hairless male and female mice (6–8 weeks old) were obtained from Charles River Laboratories (Wilmington, MA, USA). All experimental procedures were approved by the Institutional Animal Care and Use Committee (IACUC) prior to the commencement of the studies (IACUC-20631).

### 2.2. Antibodies

Monoclonal antibodies used for flow cytometry studies included anti-mouse CD11b (M1-70 PerCp), Gr-1 (RB6-8C5; AF488), CD11c (N418; APC), MHC II (M5 114.15.2; AF700), IL-12p35 (27537; PE), and IL-23p19 (fc23cpg; AF488) (Thermofisher Scientific, Waltham MA, USA).

### 2.3. UVB Light Exposure of Mice

The UV emission source (Daavlin, UVA/UVB Research Irradiation Unit, Bryan, OH, USA) consisted of four UVB lamps controlled by an electronic system to regulate UVB dosage. The mice were exposed at a fixed distance of 24 cm from the lamps to their dorsal skin surface. Wavelengths below 290 nm were filtered out using Kodacel cellulose film (Eastman Kodak Co., Rochester, NY, USA). The emitted wavelengths primarily fell within the UVB (290–320 nm; ~80%) and UVA (~20%) ranges, with peak emission observed at 314 nm, which was regularly monitored.

For all short-term experiments, mice (both male and female) were divided into four different groups: UV/honey (5 mice), UV/No honey (5 mice), No UV/honey (5 mice), No UV/No honey (5 mice). For acute UVB exposure, the mice were fed 0.1% TH in drinking water via oral gavage for 2 weeks and then exposed once to 180 mJ/cm^2^ UVB. After an incubation period of 24 h, the mice were sacrificed using isoflurane. For the carcinogenesis experiment, both male and female mice were divided into two groups, UV/honey (10 mice) and UV/No honey (10 mice). The mice were exposed to 180 mJ/cm^2^ UVB thrice weekly for 30 weeks. Mice were fed with 0.1% TH in drinking water ad libitum during the course of experiment UVB.

### 2.4. Immunohistochemistry of CPD + Cells

UVB-induced DNA damage, indicated by CPD-positive cells, was assessed using a previously established protocol with modifications [16]. In brief, frozen skin sections (5 μm thick) were thawed and treated with 70 mM NaOH in 70% ethanol for 2 min to denature nuclear DNA. This was followed by a 1 min neutralization step in 100 mM Tris-HCl (pH 7.5) in 70% ethanol. The sections were then rinsed with PBS and blocked with 10% goat serum (in PBS) before being incubated with a monoclonal anti-CPD antibody (Kamiya Biomedical Company, Seattle, WA, USA) or its isotype control (IgG1). Detection of the bound primary antibody was performed using an Alexa Fluor 488-labeled secondary antibody, and the nuclei were counterstained with DAPI. CPD-positive cells were quantified by analyzing 5–6 different fields (10× magnification) under a Keyence BZ-X710 microscope (Keyence, Itasca, IL, USA).

### 2.5. Measurement of CPD Levels Using ELISA

Skin samples from SKH-1 mice, treated with or without TH, were collected 24 h after a single UVB exposure at 180 mJ/cm^2^. Control groups were composed of mice that were not exposed to UVB or TH. After washing the skin tissue, genomic DNA was extracted using the DNeasy Blood and Tissue Kit (Qiagen, Germantown, MD, USA). CPD levels were quantified using the STA-322 DNA Damage ELISA Kit (Cell Biolabs, San Diego, CA, USA) in accordance with the manufacturer’s guidelines.

### 2.6. RNA Purification and Real-Time PCR (qPCR) Quantification

Total RNA was isolated from skin samples using Trizol reagent (Life Technologies, Carlsbad, CA, USA), following the manufacturer’s instructions. Complimentary DNA (cDNA) was synthesized from 1 µg RNA using iScript cDNA synthesis kit (Bio-Rad, Hercules, CA, USA), as per the provided protocol. Using iQ^TM^ SYBR Green Master Mix (Bio-Rad, Hercules, CA, USA), cDNA was amplified using real-time PCR with a Bio-Rad MyiQ thermocycler and SYBR Green detection system (Bio-Rad, Hercules, CA, USA). The standard PCR conditions were 95 °C for 10 min and then 40 cycles at 95 °C for 30 s, 60 °C for 30 s, and 72 °C for 30 s. The expression of *XPA* (Forward 5′-CAAAGGTGGCTTCATTTTA-3′, Reverse 5′-GGTACATGTCATCTTCTAAG-3′) was normalized to the expression level of the GAPDH mRNA in each sample. Relative mRNA expression was determined using the cycle threshold (Ct) method. The mean Ct values from duplicate measurements were used to calculate the relative expression of the target gene, normalized to the internal control, using the formula 2^−ΔΔCT^.

### 2.7. Flowcytometric Analysis Draining Lymph Nodes

Single cell suspensions were prepared from draining lymph nodes collected from groups of mice following UVB exposure, using a digestion protocol previously described [16]. The cells were stained with markers for CD11c, MHC-II, IL-23p35, IL-23p19, CD11b, and Gr-1. Flow cytometric analysis was performed using an Attune NxT flow cytometer (Thermo Fisher Scientific, Waltham, MA, USA), and data were analyzed using FlowJo software (version 10.6.1).

### 2.8. Histological Staining

SKH-1 mice, treated with or without TH, were exposed to a single UVB dose of 180 mJ/cm^2^. After 24 h, dorsal skin samples were collected, fixed in 10% formalin, and embedded in paraffin. The tissue was sectioned into 5-µm slices and stained with hematoxylin and eosin (H&E). Imaging was performed using a Keyence BZ-X710 microscope.

### 2.9. Photocarcinogenesis Study

Mice were subjected to photocarcinogenesis, as described previously [17]. The dorsal skins of SKH-1 mice (10 per group both male and female) were given 0.1% ad libitum and exposed to UVB radiation (180 mJ/cm^2^), thrice a week for up to 30 weeks. The mice received 0.1% TH or plain drinking water throughout the course of the experiment. Tumor development was monitored weekly throughout the study.

### 2.10. Statistical Analysis

For all experiments, comparisons between UVB-exposed and -unexposed groups were performed using two-way analysis of variance (ANOVA). Data are presented as mean ± SD, and a *p*-value of less than 0.05 was considered statistically significant.

## 3. Results

### 3.1. UVB-Induced DNA Damage Is Repaired in Mice Fed with TH 0.1%

**Figure 1:**
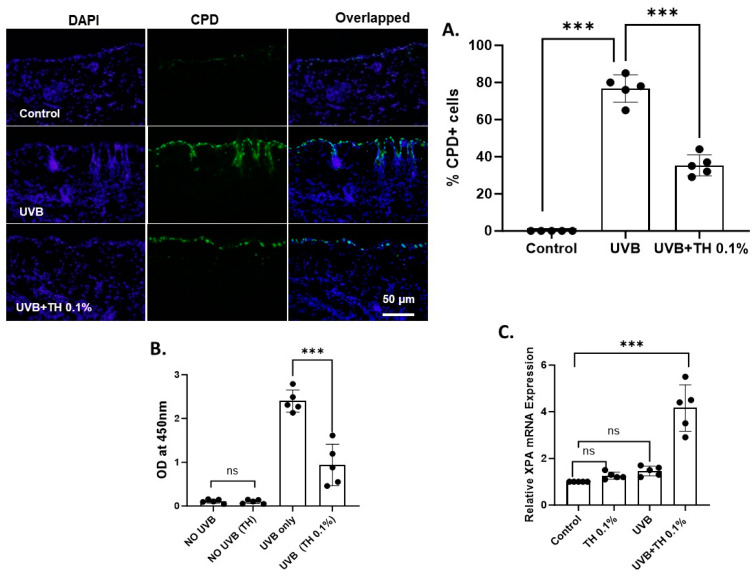
TH 0.1% augments the repair of UVB-induced CPD. (**A**) Representative images showing CPD+ cells (in green) in frozen skin sections post-UVB exposure. (**B**) ELISA quantification of CPDs. (**C**) Real-time PCR analysis of XPA mRNA expression. Data represent mean ± SD for five animals per group (*** *p* < 0.001, ns: not significant). CPD, cyclobutane pyrimidine dimer; TH, Tualang honey; UVB, ultraviolet B. Scale = 50 µM.

UVB radiation causes DNA damage in the form of CPDs, which are repaired by an NER mechanism that involving the upregulation of XPA. SKH-1 hairless mice were exposed to a single dose of UVB (180 mJ/cm^2^) after 2 weeks of daily oral gavage with 0.1% TH. There were significantly (*p =* 0.0003) fewer CPDs seen in mouse skin 24 h after exposure to UVB with 0.1% TH [Figure 1A]. This was further confirmed by ELISA [Figure 1B]. This corresponded with a significant (*p* = 0.0004) increase in XPA in mouse skin with 0.1% TH [Figure 1C].

### 3.2. TH 0.1% Augments Immunostimulatory Cytokine Production by Dendritic Cells

**Figure 2:**
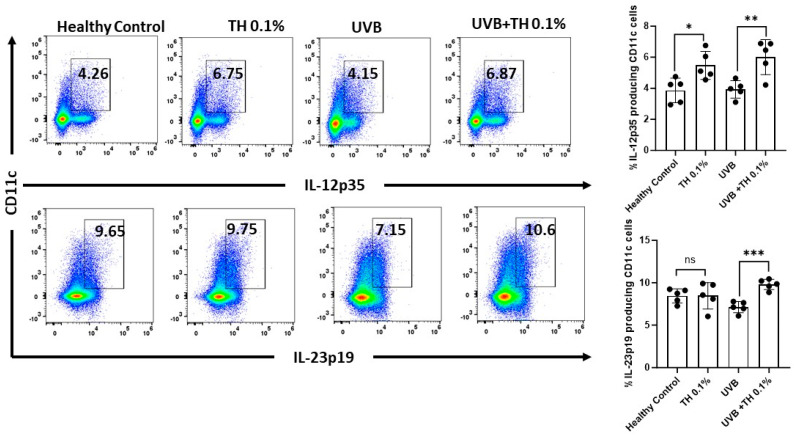
TH 0.1% increases IL-12p35- and IL-23p19-producing CD11c cells in UVB-irradiated mice. Percentages of IL-12p35- and IL-23p19-producing CD11c+ cells in draining lymph nodes were analyzed by flow cytometry 24 h post-UVB exposure. Data represent mean ± SD for five animals per group (* *p* < 0.05, ** *p* < 0.01, *** *p* < 0.001, ns: not significant). TH, tualang honey; UVB, ultraviolet B.

IL-12p35 along with IL-23p19 have been reported to stimulate the expression of DNA repair enzymes and augment the repair of UVB-induced CPDs. SKH-1 hairless mice were exposed to a single dose of UVB (180 mJ/cm^2^) after 2 weeks of daily oral gavage with 0.1% TH. Draining lymph nodes were harvested 24 h after UVB, and single-cell suspensions were formed and analyzed using flow cytometry. There was a significant (*p <* 0.001) increase in immunostimulatory cytokines IL-12p35 and IL-23p19 by CD11c+ dendritic cells in draining lymph nodes of mice fed with TH in comparison to the mice that did not receive supplementation with TH [Figure 2].

### 3.3. TH Protects Mice Against Inflammation After Acute UVB Exposure

**Figure 3:**
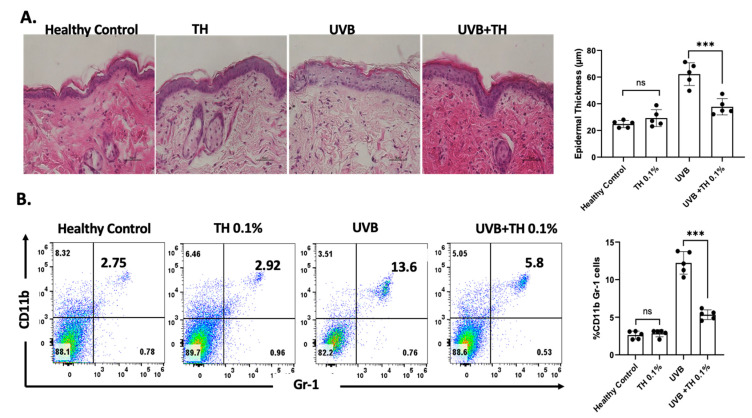
TH 0.1% decreases inflammation and CD11b+Gr-1+ myeloid cell infiltration in UVB-irradiated mice. (**A**) Representative H&E-stained skin sections showing reduced inflammation in TH-treated mice compared to controls. (**B**) Flow cytometry analysis of CD11b+Gr-1+ myeloid cells in lymph nodes. Data represent mean ± SD for five animals per group (*** *p* < 0.001, ns: not significant). TH, tualang honey; UVB, ultraviolet B. Scale = 50 µM.

SKH-1 hairless mice were exposed to a single dose of UVB radiation (180 mJ/cm^2^) after receiving 0.1% TH via daily oral gavage for 2 weeks. The dorsal skin of mice was harvested after 24 h and stained with hematoxylin and eosin. Mice that received TH had significantly less inflammation in their skin after UVB exposure in comparison to mice that received water [Figure 3A]. Mice that received TH had significantly (*p <* 0.001) fewer inflammatory CD11b+Gr-1+ myeloid cells in their skin after UVB exposure in comparison to mice that received water [Figure 3B].

### 3.4. TH Exhibits a Protective Effect Against Skin Carcinogenesis

**Figure 4:**
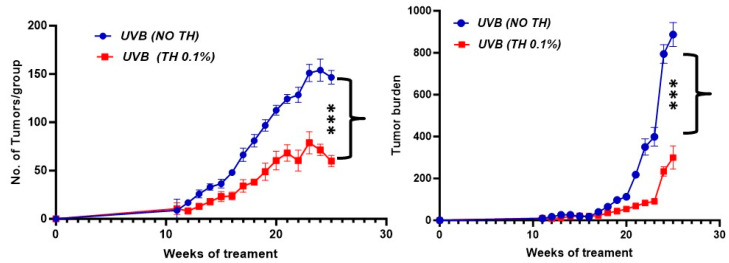
TH 0.1% inhibits tumor development in UVB-irradiated mice. SKH-1 hairless mice were exposed to UVB (180 mJ/cm^2^) thrice a week for 30 weeks. Panels of mice received 0.1% TH or plain drinking water ad libitum throughout the course of experiment. Tumor number/tumor burden were monitored weekly. Mice that received TH had significantly fewer tumors and a lower tumor burden in comparison to the mice receiving plain drinking water. Experiments were conducted in 10 mice per group. (*** *p* < 0.001). TH, tualang honey; UVB, ultraviolet B.

The SKH-1 hairless mice were exposed to UVB radiation at a dose of 180 mJ/cm^2^, three times weekly for 30 weeks, resulting in an accumulated UV dose of 16,200 mJ/cm^2^ (180 mJ/cm^2^ per exposure × (3 exposures/week × 30 weeks = 90 exposures) = 16,200 mJ/cm^2^). Mice received 0.1% TH or plain drinking water throughout the course of experiment. The number of tumors and tumor burden was enumerated in mice weekly and totaled at the end of experiment. Mice that received TH had significantly (*p <* 0.001) fewer tumors and less tumor burden in comparison to the mice receiving plain drinking water following UVB exposure [Figure 4].

## 4. Discussion

Our study provides initial evidence for the photochemopreventive potential of TH against UVB-induced DNA damage, inflammation, and skin carcinogenesis. Our findings align with previous research suggesting the protective role of natural polyphenols and phenolic acids against UV radiation-induced skin damage [13]. TH exhibits a multifaceted approach to mitigating UVB’s harmful effects by enhancing DNA repair, modulating immune responses, reducing inflammation, and reducing tumor development.

UVB induces DNA damage, primarily in the form of CPDs, which are pivotal in the initiation of nonmelanoma skin cancers [6]. Our findings demonstrate that TH significantly reduces CPDs in skin tissues, which correlates with upregulation of XPA, a critical nucleotide excision repair protein. These results are consistent with previous studies showing that dietary supplementation with natural antioxidants can enhance DNA repair mechanisms [18]. This suggests that TH may modulate NER pathways, potentially mediated by its bioactive compounds, including phenolic acids and flavonoids [14].

UVB-induced immunosuppression is a key contributor to skin carcinogenesis, as it impairs the immune system’s ability to eliminate DNA-damaged cells [3]. The observed increase in immunostimulatory cytokines IL-12p35 and IL-23p19 in TH-fed mice indicates that TH supports immune responses critical for DNA repair and tumor surveillance. IL-12 and IL-23 are known to enhance the repair of UVB-induced DNA damage and maintain T-helper-1-mediated immune responses [10,11,12]. Our data provide new insights into how dietary agents like TH can counteract UVB-induced damage by augmenting immune responses.

UVB-induced inflammation is another key factor in the pathogenesis of nonmelanoma skin cancers [19]. UVB induces generation of CD11b+Gr-1+ myeloid cells, which cause inflammation [20]. TH-fed mice exhibited significantly reduced inflammatory infiltration, as indicated by lower levels of CD11b+Gr-1+ myeloid cells, and reduced inflammation, as evidenced by histological analysis. These findings are in accordance with our previous work highlighting the anti-inflammatory properties of TH and its ability to downregulate pro-inflammatory cytokines [15]. This anti-inflammatory effect is likely mediated by the antioxidant properties of TH, which neutralize reactive oxygen species generated by UVB exposure.

The reduction in tumor incidence and burden in TH-fed mice subjected to chronic UVB exposure highlights the long-term protective effects of TH. This is consistent with previous studies that demonstrate the anticancer potential of natural antioxidants through DNA repair enhancement, immunomodulation, and inflammation reduction [13,18]. These findings suggest that TH supplementation may serve as a practical, non-invasive intervention for reducing the risk of UVB-induced skin cancers.

The protective effects of TH can be attributed to its bioactive components, including phenolic acids, flavonoids, and enzymes, which have been shown to exert antioxidant, anti-inflammatory, and immunomodulatory effects [18,21,22,23,24,25]. A total of six phenolic acids (gallic, syringic, benzoic, transcinnamic, *p*-coumaric, and caffeic acids) and five flavonoids (catechin, kaempferol, naringenin, luteolin, and apigenin) are found in TH [14,26,27,28,29]. The flavonoids not only contribute to the honey’s color, taste, and aroma but are also said to have health benefits [14]. More than half (58.5%) of TH’s composition comprises hydrocarbons [14]. TH also contains some distinct compounds that are not reported in other honeys. Some compounds found in TH previously not reported in other honeys are stearic acids, 2-cyclopentene-1,4,-dione, 2[3H]-furanone or dihydro-butyrolactone, gamma-crotonolactone or 2[5H]-furanone, 2-hydroxy-2-cyclopenten-1-one, hyacinthin, 2,4-dihydroxy-2,5-dimethyl-3[2H]-furan-3-one, and phenylethanol [14,26,27]. The enhancement of DNA repair and cytokine production observed in this study aligns with the known effects of these compounds. Importantly, the results highlight the potential of TH as a dietary supplement for skin cancer prevention, especially in populations at high risk of UVB exposure.

While our study demonstrates the protective effects of TH, further research is needed to elucidate the molecular pathways underlying its activity. Studies should investigate the role of individual bioactive components in modulating DNA repair and immune responses. Additionally, human clinical trials are necessary to confirm the translational potential of TH and determine optimal dosing and delivery methods. Comparative studies with other photochemopreventive agents could also help contextualize its efficacy.

Poor oral bioavailability has been a major limitation for the successful use of dietary flavonoids as cancer chemopreventive agents. Pharmacokinetic studies are needed to assess the bioavailability of TH. Furthermore, future studies with bioactive compounds of TH are warranted.

This study establishes tualang honey as a promising photochemopreventive agent that mitigates UVB-induced DNA damage, reduces inflammation, and protects against skin carcinogenesis. Its multifaceted protective effects, coupled with its natural origin and safety profile, make it an attractive candidate for further investigation in skin-cancer-prevention strategies.

## 5. Conclusions

Oral administration of TH resulted in fewer UVB-induced CPDs in skin due to enhanced repair process mediated by XPA in skin after single exposure. The immunostimulatory cytokines IL-12 and IL-23 were also upregulated in CD11c dendritic cells in mice from the TH group. There was significantly less inflammation in skin in mice when mice with TH were exposed to a single dose of UVB. Finally, mice that consumed TH were also protected against UVB-induced skin carcinogenesis. Overall, our study provides initial evidence for protective effect of TH against UVB-induced DNA damage, inflammation, and skin carcinogenesis.

## Data Availability

The original contributions presented in this study are included in the article. Further inquiries can be directed to the corresponding author.
